# Data gaps affecting health-related sustainable development goal indicators 

**DOI:** 10.2471/BLT.25.294190

**Published:** 2026-04-02

**Authors:** Keyrellous Adib, Nagui Salama, Nicholas Letchford, Stefania Davia, Natasha Azzopardi-Muscat, Hans Henri P Kluge, David Novillo-Ortiz

**Affiliations:** a Health Systems Department, World Health Organization Regional Office for Europe, UN City, Marmorvej 51, DK-2100 Copenhagen, Denmark.; b World Health Organization Regional Office for Europe, Copenhagen, Denmark.

## Abstract

**Objective:**

To evaluate the extent and distribution of missing data across health-related sustainable development goal (SDG) indicators in World Health Organization (WHO) regions, over time and by disaggregation and method of data collection.

**Methods:**

We conducted a descriptive cross-sectional analysis of health-related indicators from the United Nations SDG indicators database. Our analysis included data from 194 Member States between 2015 and 2024. We assessed the overall proportion of missing data by WHO region, variation over time, level of disaggregation, data timeliness and methods.

**Findings:**

We observed pronounced data gaps and inconsistencies across WHO regions and over time. At the target level, about one third of targets (8 out of 27) had over 90% missing data points, while 41 of 43 indicators had more than 90% missing data in 2024, compared with 11 indicators in 2019. At least one form of disaggregation was present in 72% of indicators, yet missing data did not vary significantly by disaggregation level. Across regions, the number of indicators with over 90% missing data ranged from 12 to 16. Methodological differences also influenced data availability: indicators relying on estimates had substantially higher coverage than those based solely on empirical data; 49% relied on estimates, 35% on empirical data and 16% on a combination of both.

**Conclusion:**

This study underscores critical limitations in the availability, timeliness and consistency of health-related SDG data across WHO regions, highlighting the need for strengthened data systems to support the monitoring of global health progress.

## Résumé

**Objectif:**

Évaluer l’ampleur et la répartition des données manquantes parmi les indicateurs des objectifs de développement durable (ODD) liés à la santé dans les différentes régions de l’Organisation mondiale de la Santé (OMS), au fil du temps et selon le niveau de désagrégation et la méthode de collecte des données.

**Méthodes:**

Nous avons mené une analyse descriptive transversale des indicateurs liés à la santé issus du portail des données des indicateurs des ODD des Nations Unies. Notre analyse comprenait les données de 194 États membres entre 2015 et 2024. Nous avons évalué la proportion globale de données manquantes par région de l’OMS, la variation dans le temps, le niveau de désagrégation, l’actualité des données et les méthodes utilisées.

**Résultats:**

Nous avons constaté des lacunes et des incohérences marquées dans les données entre les régions de l’OMS et au fil du temps. Environ un tiers des cibles (8 sur 27) présentaient plus de 90% de données manquantes, tandis que 41 des 43 indicateurs affichaient plus de 90% de données manquantes en 2024, contre 11 indicateurs en 2019. Au moins une forme de désagrégation était présente dans 72% des indicateurs, mais les données manquantes ne variaient pas de manière significative selon le niveau de désagrégation. Dans l’ensemble des régions, entre 12 et 16 indicateurs présentaient plus de 90% de données manquantes. Des différences méthodologiques ont également influencé la disponibilité des données: les indicateurs s’appuyant sur des estimations présentaient une couverture nettement supérieure à ceux reposant uniquement sur des données empiriques; 49% s’appuyaient sur des estimations, 35% sur des données empiriques et 16% sur une combinaison des deux.

**Conclusion:**

Cette étude met en évidence des limites critiques en matière de disponibilité, d’actualité et de cohérence des données relatives aux ODD liés à la santé dans les différentes régions de l’OMS, soulignant la nécessité de renforcer les systèmes de données afin de soutenir le suivi des progrès mondiaux en matière de santé.

## Resumen

**Objetivo:**

Evaluar la magnitud y la distribución de los datos faltantes en los indicadores de los Objetivos de Desarrollo Sostenible (ODS) relacionados con la salud en las regiones de la Organización Mundial de la Salud (OMS), con el paso del tiempo y según el nivel de desagregación y el método de recopilación de datos.

**Métodos:**

Se realizó un análisis descriptivo transversal de los indicadores relacionados con la salud de la base de datos de indicadores de los ODS de las Naciones Unidas. El análisis incluyó datos de 194 Estados Miembros entre 2015 y 2024. Se evaluó la proporción global de datos faltantes por región de la OMS, su variación con el paso del tiempo, el nivel de desagregación, la oportunidad de los datos y los métodos de recopilación.

**Resultados:**

Se observaron importantes lagunas de datos e inconsistencias entre las regiones de la OMS y con el paso del tiempo. A nivel de metas, aproximadamente un tercio de ellas (8 de 27) presentaban más del 90% de datos faltantes, mientras que 41 de 43 indicadores tenían más del 90% de datos faltantes en 2024, en comparación con 11 indicadores en 2019. Al menos una forma de desagregación estaba presente en el 72% de los indicadores; sin embargo, los datos faltantes no variaron significativamente según el nivel de desagregación. Entre regiones, el número de indicadores con más del 90% de datos faltantes osciló entre 12 y 16. Las diferencias metodológicas también influyeron en la disponibilidad de datos: los indicadores basados en estimaciones presentaron una cobertura sustancialmente mayor que aquellos basados exclusivamente en datos empíricos; el 49% se basaban en estimaciones, el 35% en datos empíricos y el 16% en una combinación de ambos.

**Conclusión:**

Este estudio destaca las limitaciones críticas en la disponibilidad, la oportunidad y la consistencia de los datos de los ODS relacionados con la salud en las regiones de la OMS, lo que subraya la necesidad de fortalecer los sistemas de datos para apoyar el seguimiento de los progresos en salud a nivel mundial.

## ملخص

**الغرض:**

تقييم مدى وتوزيع البيانات المفقودة في مؤشرات أهداف التنمية المستدامة المتعلقة بالصحة (SDG) في أقاليم منظمة الصحة العالمية، على مر الزمن وحسب مستوى التفصيل وطريقة جمع البيانات.

**الطريقة:**

قمنا بإجراء تحليل وصفي مقطعي للمؤشرات المتعلقة بالصحية من قاعدة بيانات مؤشرات أهداف التنمية المستدامة التابعة للأمم المتحدة. شمل تحليلنا بيانات من 194 دولة عضوًا خلال الفترة من 2015 إلى 2024. وقمنا بتقييم النسبة الإجمالية للبيانات المفقودة حسب إقليم منظمة الصحة العالمية، والتغيرات عبر الزمن، ومستوى التفصيل، وتوقيت جمع البيانات، والأساليب المتبعة.

**النتائج:**

لاحظنا وجود فجوات وتناقضات واضحة في البيانات بين أقاليم منظمة الصحة العالمية وعلى مر الزمن. على مستوى الأهداف، احتوى حوالي ثلث الأهداف (8 من أصل 27) على بيانات مفقودة بنسبة تزيد عن %90، بينما احتوى 41 مؤشرًا من أصل 43 على بيانات مفقودة بنسبة تزيد عن %90 في عام 2024، مقارنةً بـ 11 مؤشرًا في عام 2019. تم العثور على شكل واحد على الأقل من أشكال التفصيل في %72 من المؤشرات، إلا أن البيانات المفقودة لم تختلف اختلافًا كبيرًا باختلاف مستوى التفصيل. وعلى مستوى المناطق، تراوح عدد المؤشرات التي تحتوي على بيانات مفقودة بنسبة تزيد عن %90 بين 12 و16 مؤشرًا. كما أثرت الاختلافات المنهجية على توافر البيانات: إذ حظيت المؤشرات التي تعتمد على التقديرات بتغطية أعلى بكثير من تلك التي تعتمد فقط على البيانات التجريبية؛ حيث اعتمد %49 على التقديرات، و%35 على البيانات التجريبية، و%16 على مزيج من الاثنين.

**الاستنتاج:**

تؤكد هذه الدراسة على وجود قيود أساسية في توافر بيانات أهداف التنمية المستدامة المتعلقة بالصحة، وتوقيتها، واتساقها في جميع مناطق منظمة الصحة العالمية، مما يُسلط الضوء على الحاجة إلى تعزيز أنظمة البيانات لدعم رصد التقدم الصحي العالمي.

## 摘要

**目的:**

旨在基于不同时期、细分程度以及数据收集方法来评估世卫组织 (WHO) 各个区域内健康相关可持续发展目标 (SDG) 指标中缺失数据所占比例及其分布情况。

**方法:**

我们对从联合国 SDG 指标数据库中获取的健康相关指标进行了描述性横剖分析。我们将 2015 年至 2024 年期间 194 个成员国的相关数据纳入了分析范畴。我们基于世卫组织的各个区域、不同时期的变化、细分程度、数据及时性和数据收集方法评估了缺失数据的总体比例。

**结果:**

我们发现，世卫组织各个区域以及不同时期之间明显存在数据差距和不一致性。在目标层面，大约三分之一目标（8 个，共 27 个）的缺失数据点所占比例超过了 90%，而在指标层面，2024 年，43 个指标中有 41 个的缺失数据所占比例超过了 90%，相比之下，2019 年仅有 11 个指标存在这种情况。我们对 72% 的指标至少进行了一次细分，但缺失数据并未因细分程度不同而发生显著变化。世卫组织各个区域缺失数据所占比例超过 90% 的指标数量分别为 12 到 16 个不等。方法学差异也影响了数据的可用性：基于估算数据的指标的覆盖率明显高于那些仅基于实证数据的指标；其中 49% 的指标基于估算数据，35% 基于实证数据，剩余 16% 则同时基于估算数据和实证数据。

**结论:**

本项研究揭示了世卫组织各个区域在健康相关 SDG 数据可用性、及时性和一致性方面的重大缺陷，从而凸显了强化数据系统以支持实施全球健康进展监测的必要性。

## Резюме

**Цель:**

Дать оценку масштаба и распределения отсутствующих данных по показателям целей в области устойчивого развития (ЦУР) в сфере здравоохранения в регионах Всемирной организации здравоохранения (ВОЗ) с течением времени, а также с разбивкой по дезагрегации и методам сбора данных.

**Методы:**

Авторы провели дескриптивный перекрестный анализ базы данных показателей ЦУР Организации Объединенных Наций в отношении связанных со здравоохранением показателей. В анализ были включены данные 194 государств-членов за период с 2015 по 2024 год. Были оценены: общая доля отсутствующих данных по каждому региону ВОЗ, изменения во времени, уровень детализации данных, их своевременность и методы сбора.

**Результаты:**

Наблюдались выраженные пробелы в данных и неоднородность как между регионами ВОЗ, так и во времени. На уровне целей примерно до трети из них (8 из 27) демонстрировали нехватку данных до 90%, при этом для 41 из 43 показателей в 2024 году отсутствовало более 90% данных (для сравнения: в 2019 году таких было всего 11). По меньшей мере одна форма дезагрегации присутствовала в 72% показателей, однако доля отсутствующих данных не различалась существенно в зависимости от уровня дезагрегации. В разных регионах количество показателей с отсутствием более чем 90% данных варьировалось от 12 до 16. Методологические различия также влияли на доступность данных: опирающиеся на оценку показатели имели значительно более широкий охват, нежели те, которые опирались только на эмпирические данные (49% опирались на данные оценки, 35% – на эмпирические данные, 16% – на те и другие одновременно).

**Вывод:**

Исследование подчеркивает существенные ограничения в доступности, своевременности и согласованности данных по показателям ЦУР в сфере здравоохранения в регионах ВОЗ, указывая на необходимость укрепления систем данных для поддержки мониторинга глобального прогресса в области здравоохранения.

## Introduction

The concept of health-related sustainable development goals (SDGs) is used to capture the multidimensional nature of health within the United Nations (UN) *Transforming our world: the 2030 agenda for sustainable development*.^1^ The health-related goals are primarily reflected in SDG 3, that is ensure healthy lives and promote well-being for all at all ages. While there are conceptual linkages between SDG 3 and the remaining sixteen goals,^2^ determining which targets and indicators across those other goals should be classified as health-related is more challenging. Different sources monitor different sets of targets and indicators.^3^^–^^5^ This variation reflects the judgment required to define health-related indicators; it includes assessing an indicator’s proximity to health outcomes, and its scope within the health system's direct or stewardship functions for providing health interventions or reducing health risks.^6^

The World Health Organization (WHO) is mandated by its Member States to compile and disseminate health statistics;^7^ however, delayed or incomplete data can undermine WHO’s technical support to Member States. Up to 57 indicators were acknowledged as health-related indicators.^6^^,^^8^ From these, the Organization’s *Thirteenth general programme of work, 2019–2025* included 39 SDG indicators, 26 from SDG 3 and 13 from other goals; in addition seven indicators relating to World Health Assembly resolutions were included in the programme.^9^ In the *Draft fourteenth general programme of work* (GPW 14), these indicators were retained and mapped onto new outcomes.^10^^,^^11^

Accurate, complete and timely data are essential for effective global health governance. They support the setting of clear goals, monitoring progress and priority-setting, as well as strengthening accountability through transparent reporting. Data are also important for identifying and addressing inequalities. Disaggregated data by age, sex, socioeconomic status and geographic location reveal disparities in health outcomes and can support targeted interventions to address the needs of vulnerable populations. Comparable data enable analyses across regions, populations and time. Robust data systems inform decision-making and guide the efficient and impactful allocation of resources and funding by strengthening the understanding of health challenges and trends. This approach contributes to improved health outcomes and a more equitable distribution of health services.^6^^,^^11^^–^^13^

WHO plays a critical role in adjusting, validating and reporting statistics used to monitor global health indicators. Unadjusted statistics, which may have missing data and are derived directly from primary data without corrections, are valuable for public health responses but have limitations. In contrast, adjusted estimates use techniques like imputation to fill data gaps and improve comparability. Further, advanced methods can synthesize data from multiple sources and adjust empirical data using statistical models. However, discrepancies in reported indicator estimates can still arise due to differing estimation methods, a preference for unadjusted data, the availability of new data or disagreements over variables. To ensure consistency, WHO collaborates with other UN agencies to combine technical resources and provide unified estimates for various health-related targets.^14^^,^^15^

While measuring the progress towards all the SDGs is constrained by limitations in data availability and timeliness, substantial improvements have been made in developing internationally comparable monitoring data. In 2016, about one third of indicators had data coverage and two fifths lacked standardized methods. By 2024, two thirds of indicators had good coverage and all 231 indicators had well-established and internationally agreed methods. Good trend data were available for about half of the indicators across more than half of all countries.^13^ However, the GPW 14 results framework acknowledges that tracking the health-related SDGs presents challenges due to incomplete data and slow progress towards goals.^11^

Over halfway through the 2030 agenda for sustainable development,^16^ we aimed to evaluate the extent and distribution of missing health-related SDG indicators across WHO regions, over time and by disaggregation and data collection method. We reviewed indicators from the GPW 14 results framework to reflect core global health priorities and to support more consolidated global reporting of health-related goals.^11^ The framework includes 98 proposed outcome indicators; for this analysis, we focused on 43 health-related SDG indicators that represent progress towards WHO’s Triple Billion targets. These targets are a strategic framework to accelerate progress towards the SDGs through universal health coverage, protection from health emergencies and healthier populations.^17^ Within the framework, these health-related SDG indicators can be counted as 46, however, three entries correspond to subcomponents of the same SDG indicator or share identical SDG indicator numbering, despite being listed separately.^11^ To avoid double counting while maintaining full analytical coverage, we consolidated these into 43 analytically distinct indicators.

## Methods

### Data source

As of 9 September 2025, we extracted data on the selected indicators from the UN SDG indicators database for all 194 WHO Member States.^18^^,^^19^ The database monitors global SDG progress and covers over 210 indicators. By aggregating data from SDG and continental regions, countries and areas, the database enables users to track trends, compare performance and identify areas for improvement. 

### Health-related SDG indicators

Our analysis included 43 health-related indicators (Table 1). All SDG indicators are classified by the Inter-agency and Expert Group on SDG Indicators (IAEG-SDGs) into two tiers based on global data availability. Both tiers have clear concepts and established methods, however, Tier I indicators have regular data production covering at least 50% of countries and of the population in every region where the indicator is relevant, while Tier II indicators lack regular data production by countries.^20^

**Table 1:** Health-related SDG indicators included in the study on the extent and distribution of missing indicator data

Target	Indicator description	Disaggregation available in SDG database	Tier classification, I or II^a^
**1.1 By 2030, eradicate extreme poverty for all people everywhere, currently measured as people living on less than US$ 1.25 a day**			
1.1.1	Proportion of the population living below the international poverty line by sex, age, employment status and geographic location (urban/rural)^b^	NA	NA
**1.3 Implement nationally appropriate social protection systems and measures for all, including floors, and by 2030 achieve substantial coverage of the poor and the vulnerable**			
1.3.1	Proportion of population covered by at least one social protection benefit (%)	Age, sex, quantile, employment, recipient, type of benefit	I
**2.2 By 2030, end all forms of malnutrition, including achieving, by 2025, the internationally agreed targets on stunting and wasting in children under 5 years of age and address the nutritional needs of adolescent girls, pregnant and lactating women and older persons**			
2.2.1	Prevalence of stunting (height for age < −2 standard deviation from the median of WHO Child Growth Standards) among children under 5 years of age	Sex and unit of measurement	I
2.2.2	Prevalence of overweight (weight for height > +2 standard deviation from the median of WHO Child Growth Standards) and prevalence of wasting (weight for height < −2 standard deviation from the median of WHO Child Growth Standards) among children under 5 years of age	Sex, unit of measurement and health outcome	I
2.2.3	Prevalence of anaemia in women aged 15 to 49 years, by pregnancy status (%)	Pregnancy status	I
**3.1 By 2030, reduce the global maternal mortality ratio to less than 70 per 100 000 live births**			
3.1.1	Maternal mortality ratio	NA	I
3.1.2	Proportion of births attended by skilled health personnel	NA	I
**3.2 By 2030, end preventable deaths of newborns and children under 5 years of age, with all countries aiming to reduce neonatal mortality to at least as low as 12 per 1000 live births and under-5 mortality to at least as low as 25 per 1000 live births**			
3.2.1	Under-5 mortality rate	Age, sex and unit of measurement	I
3.2.2	Neonatal mortality rate	Unit of measurement	I
**3.3. By 2030, end the epidemics of AIDS, tuberculosis, malaria and neglected tropical diseases and combat hepatitis, waterborne diseases and other communicable diseases**			
3.3.1	Prevalence of active syphilis in individuals 15 to 49 years of age (%) (new) and the number of new HIV infections per 1000 uninfected population, by sex, age and key populations (GPW 13)	Age and sex	I
3.3.2	Tuberculosis incidence per 100 000 population	NA	I
3.3.3	Malaria incidence per 1000 population	NA	I
3.3.4	Hepatitis B incidence per 100 000 population	NA	I
3.3.5	Number of people requiring interventions against neglected tropical diseases	NA	I
**3.4 By 2030, reduce by one third premature mortality from noncommunicable diseases through prevention and treatment and promote mental health and well-being**			
3.4.1	Mortality rate attributed to cardiovascular disease, cancer, diabetes or chronic respiratory disease	Sex, name of non-communicable disease and unit of measurement	I
3.4.2	Suicide mortality rate	Sex	I
**3.5 Strengthen the prevention and treatment of substance abuse, including narcotic drug abuse and harmful use of alcohol**			
3.5.1	Coverage of treatment interventions (pharmacological, psychosocial and rehabilitation and aftercare services) for substance use disorders	Sex and substance use disorders	II
3.5.2	Alcohol per capita consumption (aged 15 years and older) within a calendar year in litres of pure alcohol	NA	I
**3.6 By 2030, halve the number of global deaths and injuries from road traffic accidents**			
3.6.1	Death rate due to road traffic injuries	Unit of measurement	I
**3.7 By 2030, ensure universal access to sexual and reproductive health-care services, including for family planning, information and education and the integration of reproductive health into national strategies and programmes**			
3.7.1	Proportion of women of reproductive age (aged 15–49 years) who have their need for family planning satisfied with modern methods	NA	I
3.7.2	Adolescent birth rate (aged 10–14 years; aged 15–19 years) per 1000 women in that age group	Age	I
**3.8 Achieve universal health coverage, including financial risk protection, access to quality essential health-care services and access to safe, effective, quality and affordable essential medicines and vaccines for all**			
3.8.1	Coverage of essential health services	NA	I
3.8.2	Incidence of catastrophic out-of-pocket health spending	Expenditure threshold	I
**3.9 Substantially reduce the number of deaths and illnesses from hazardous chemicals and air, water and soil pollution and contamination**			
3.9.1	Mortality rate attributed to household and ambient air pollution	Source of pollution	I
3.9.2	Mortality rate attributed to unsafe water, unsafe sanitation and lack of hygiene (exposure to unsafe Water, Sanitation and Hygiene for All [WASH] services)	NA	I
3.9.3	Mortality rate attributed to unintentional poisoning	Sex	I
**3.a Strengthen the implementation of the World Health Organization Framework Convention on Tobacco Control in all countries, as appropriate**			
3.a.1	Age-standardized prevalence of current tobacco use among persons aged 15 years and older	Sex	I
**3.b Support the research and development of vaccines and medicines for the communicable and noncommunicable diseases that primarily affect developing countries, provide access to affordable essential medicines and vaccines, in accordance with the Doha Declaration on the TRIPS Agreement and Public Health, which affirms the right of developing countries to use to the full the provisions in the Agreement on Trade-Related Aspects of Intellectual Property Rights regarding flexibilities to protect public health and, in particular, provide access to medicines for all**			
3.b.1	Proportion of the target population covered by all vaccines included in their national programme	Vaccine regimen	I
**3.c Substantially increase health financing and the recruitment, development, training and retention of the health workforce in developing countries, especially in least-developed countries and small island developing States**			
3.c.1	Health worker density and distribution (by occupation, subnational, facility ownership, facility type, age group, sex)	Sex, type of occupation and unit of measurement	I
**3.d Strengthen the capacity of all countries, in particular developing countries, for early warning, risk reduction and management of national and global health risks**			
3.d.1	International Health Regulations (IHR) (2005) capacity and health emergency preparedness	Type of IHR capacity	I
3.d.2	Percentage of bloodstream infections due to selected antimicrobial-resistant organisms	Resistant pathogen	II
**4.2 By 2030, ensure that all girls and boys have access to quality early childhood development, care and pre-primary education so that they are ready for primary education**			
4.2.1	Proportion of children aged 24–59 months who are developmentally on-track in health, learning and psychosocial well-being, by sex	Sex and development domains	II
**5.2 Eliminate all forms of violence against all women and girls in the public and private spheres, including trafficking and sexual and other types of exploitation**			
5.2.1	Proportion of ever-partnered women and girls aged 15 years and older subjected to physical, sexual or psychological violence by a current or former intimate partner in the previous 12 months, by form of violence and by age	Age	I
**5.3 Eliminate all harmful practices, such as child, early and forced marriage and female genital mutilation**			
5.3.2	Proportion of girls and women aged 15–49 who have undergone female genital mutilation	NA	I
**5.6 Ensure universal access to sexual and reproductive health and reproductive rights as agreed in accordance with the Programme of Action of the International Conference on Population and Development and the Beijing Platform for Action and the outcome documents of their review conferences**			
5.6.1	Proportion of women aged 15–49 years who make their own informed decisions regarding sexual relations, contraceptive use and reproductive health care	Decision-making domain	II
5.6.2	Number of countries with laws and regulations that guarantee full and equal access to women and men aged 15 years and older to sexual and reproductive health care, information and education	Policy area/component	I
**6.1 By 2030, achieve universal and equitable access to safe and affordable drinking water for all**			
6.1.1	Proportion of population using safely managed drinking water services	Location	I
**6.2 By 2030, achieve access to adequate and equitable sanitation and hygiene for all and end open defecation, paying special attention to the needs of women and girls and those in vulnerable situations**			
6.2.1	Proportion of population using (a) safely managed sanitation services and (b) a hand-washing facility with soap and water	Location and sanitation and hygiene service type	I(a) and II (b)
**7.1 By 2030, ensure universal access to affordable, reliable and modern energy services**			
7.1.2	Proportion of population with primary reliance on clean fuels and technology	Location	I
**10.7 Facilitate orderly, safe, regular and responsible migration and mobility of people, including through the implementation of planned and well-managed migration policies**			
10.7.2	Does the government provide non-national (including refugees and migrants) equal access to (i) essential and/or (ii) emergency health care	Policy domains and unit of measurement	I
**11.1 By 2030, ensure access for all to adequate, safe and affordable housing and basic services and upgrade slums**			
11.1.1	Proportion of urban population living in slums, informal settlements or inadequate housing	Cities, housing condition and location	I
**11.6 By 2030, reduce the adverse per capita environmental impact of cities, including by paying special attention to air quality and municipal and other waste management**			
11.6.2	Annual mean levels of fine particulate matter (e.g. PM2.5 and PM10) in cities (population weighted)	Location	I
**16.2 End abuse, exploitation, trafficking and all forms of violence against and torture of children**			
16.2.1	Proportion of children aged 1–17 years who experienced any physical punishment and/or psychological aggression by caregivers in the past month	NA	II

NA: not applicable; SDG: sustainable development goal; UN: United Nations.

^a^ Tier 1 and Tier 2 are classifications for SDG indicators used by the UN Statistical Commission to monitor data availability. Tier 1 indicators have established methodologies and are widespread, regular data. Tier 2 indicators have established methodologies but lack regular, consistent data collection across countries.

^b^ While this indicator is not directly covered in WHO’s GPW 14 results framework, another related indicator (Incidence of impoverishing out-of-pocket health spending) is included, referring to the relationship with this SDG indicator (1.1.1).

Notes: NA indicates that no disaggregation is available in the UN SDG indicators database portal. The units of measurement include numbers, percentages (%) and/or per capita counts. More detailed metadata on the items under each disaggregation category can be found in the UN SDG indicators database.^18^ Indicator and indicator description is verbatim from https://unstats.un.org/sdgs/indicators/Global-Indicator-Framework-after-2025-review-English.pdf.

Indicators may be disaggregated, including but not limited to sex, age, unit of measurement, location and quartile. For some indicators, the disaggregation labels are either inconsistent with their definitions or redundant. To improve consistency and reduce redundancy, we updated the labels to align with their definitions, as follows:

Indicator 11.1.1 has 47 disaggregations, including location, housing condition and city. As the city disaggregation is specific to a single country, we converted it to a binary value (either “city” or “no city”). This change leaves only 7 disaggregations for indicator 11.1.1. in our analysis.

Indicator 3.d.1 has 41 disaggregations, all of which relate to the capacity to comply with the International Health Regulations (IHR)(2005). These are broken down as follows: 13 cover the period 2015–2017 and are named IHR (1–13); another 13 cover the period 2018–2020 and are named States Parties Self-Assessment Annual Report (SPAR) (1–13); lastly, 15 focus on the period 2021–2023 and have the name SPAR2-C (1–15) which refers to the second edition of States Parties Self-Assessment Annual Report. Despite these name changes, their specific definitions have remained consistent across the years, except for the last two disaggregations (SPAR2-C14 and SPAR2-C15).^18^ Because the definitions for these disaggregations are consistent, we renamed them to match their definitions. Specifically, those previously labelled IHR (1–13) and SPAR (1–13) are now called SPAR2-C (1–13). The disaggregations of SPAR2-C14 and SPAR2-C15 remain unchanged.

Two indicators include age groups that are inconsistent with their definitions. Indicator 4.2.1 focuses on child development and has two disaggregations, each with a definition that focuses on distinct yet separate age groups: 36–59 months old and 24–59 months old. Similarly, indicator 16.2.1 focuses on abuse of children aged 1–14 years old, but several countries used their own age definitions for these indicators. The labels for 4.2.1 and 16.2.1 were therefore modified to align with the definitions provided.^18^

After the modifications to indicators 11.1.1 and 3.d.1, the indicators with the most disaggregations are 3.4.1 with 45 disaggregations and 1.3.1 with 31 disaggregations. There are 12 indicators with no disaggregations.

### Data processing

The downloaded data included rows for each indicator, country and year where a numeric value was available. However, there were data gaps between years (for specific countries and indicators).^18^ To calculate the percentage of missing data among the indicators, we first needed to ensure that all potential data points were explicitly represented, so that missingness could be measured consistently across indicators, countries and years. To achieve this, we constructed a complete reference structure of all possible data points by generating a data set listing every possible combination of year, indicator (including disaggregations) and country. This data set initially contained no values, hence we describe it as empty, but it provided the full set of rows against which observed data could be compared.

We considered indicators complete if they contained a numeric value for each country for each year from 2015 to 2024, covering all years for which data were recorded.^21^ We then merged the empty reference data set with the UN SDG indicators database to align the observed values with the complete set of expected rows. After merging, we cleaned any duplicate rows (where both a numeric value and a placeholder NA [not available] appeared for the same indicator, country and year) by retaining the numeric entry and dropping the placeholder. This process ensured that the data set contained one row for every possible combination of indicator, country and year. Rows with numeric values were counted as observed data and rows retaining NA were treated as missing.

To facilitate regional comparisons, the indicators at the Member State level were aggregated into the six WHO regions: African Region, Region of the Americas, South-East Asia Region, European Region, Eastern Mediterranean Region and Western Pacific Region. The Member States are listed by region in the online repository.^22^

### Data analysis

We conducted a descriptive cross-sectional analysis to assess the percentage of missing data of health-related SDG indicators. Results are presented by region, by the method used to calculate the indicators (estimates vs empirical data) and over time. The detailed distribution of missing data is described in the online repository.^22^ We conducted a Pearson correlation test between the number of disaggregations per indicator and the percentage of missing data, speculating that indicators with more disaggregations were more likely to have greater percentages of missing data. Based on these observations, we identified regions and indicators that need further improvement. We used the software R, version 4.3.2 (R Foundation, Vienna, Austria), to conduct all analyses and visualizations.

## Results

### Distribution of missing data 

The 43 health-related indicators span 27 SDG targets, and our analysis revealed notable variability in data availability across those targets. Table 2 shows the proportion of data missing for each target, aggregated across all Member States and years. Notably, a substantial portion of the targets exhibited very high data gaps. Specifically, eight of 27 targets had more than 90% of missing data points. In contrast, only one, Target 3.2, which focuses on infant and under-5 mortality, had only 10% (2716/27 160) missing data.

**Table 2:** Proportion of missing data across health-related SDG indicators, 2015–2024

Target, indicator^a^	No. of subindicators^b^	Country-year combinations^c^	
		Expected, no.	Missing, no. (%)
**1.1 International poverty**			
1.1.1	17	50 440	44 669 (89)
**1.3 Social protection**			
1.3.1	31	60 140	52 548 (87)
**2.2 Malnutrition**			
2.2.1	6	11 640	2 040 (18)
2.2.2	8	15 520	5 242 (34)
2.2.3	3	5 820	609 (11)
Total	17	32 980	7 891 (24)
**3.1 Maternal mortality**			
3.1.1	1	1 940	203 (11)
3.1.2	1	1 940	1 202 (62)
Total	2	3 880	1 405 (36)
**3.2 Child mortality**			
3.2.1	12	23 280	2 328 (10)
3.2.2	2	3 880	388 (10)
Total	14	27 160	2 716 (10)
**3.3. Communicable diseases**			
3.3.1	13	50 440	34 514 (68)
3.3.2	1	1 940	194 (10)
3.3.3	1	7 760	6 797 (88)
3.3.4	1	1 940	1 652 (85)
3.3.5	1	1 940	194 (10)
Total	17	64 020	43 351 (68)
**3.4 Noncommunicable diseases and mental health**			
3.4.1	3	17 460	15 264 (87)
3.4.2	3	17 460	15 264 (87)
Total	6	34 920	30 528 (87)
**3.5 Substance use disorder**			
3.5.1	16	46 560	44 255 (95)
3.5.2	1	3 880	2 376 (61)
Total	17	50 440	46 631 (92)
**3.6 Road traffic accidents**			
3.6.1	2	11 640	11 254 (97)
**3.7 Sexual and reproductive health**			
3.7.1	1	1 940	1 759 (91)
3.7.2	2	7 760	5 853 (75)
Total	3	9 700	7 612 (78)
**3.8 Universal health coverage**			
3.8.1	1	1 940	1 164 (60)
3.8.2	2	11 640	11 098 (95)
Total	3	13 580	12 262 (90)
**3.9 Health impact of pollution**			
3.9.1	3	11 640	11 094 (95)
3.9.2	1	1 940	1 757 (91)
3.9.3	3	17 460	15 264 (87)
Total	7	31 040	28 115 (91)
**3.a Tobacco control**			
3.a.1	3	5 820	4 335 (75)
**3.b Immunization coverage**			
3.b.1	4	13 580	8 174 (60)
**3.c Health workforce**			
3.c.1	8	15 520	8 704 (56)
**3.d Management of health risks**			
3.d.1	15	29 100	8 430 (29)
3.d.2	2	3 880	3 072 (79)
Total	17	32 980	11 502 (35)
**4.2 Early childhood education and development**			
4.2.1	6	23 280	23 036 (99)
**5.2 Violence against women and girls**			
5.2.1	2	3 880	3 570 (92)
**5.3 Child marriage and female genital mutilation**			
5.3.2	1	3 880	3 855 (99)
**5.6 Reproductive health access and rights**			
5.6.1	4	7 760	7 476 (96)
5.6.2	18	34 920	32 349 (93)
Total	22	42 680	39 825 (93)
**6.1 Safe drinking water**			
6.1.1	3	5 820	3 523 (61)
**6.2 Access to sanitation and hygiene**			
6.2.1	9	17 460	8 559 (49)
**7.1 Access to energy services**			
7.1.2	3	7 760	2 603 (34)
**10.7 Safe migration and mobility**			
10.7.2	7	13 580	12 187 (90)
**11.1 Housing**			
11.1.1	6	13 580	11 897 (88)
**11.6 Urban environmental impact**			
11.6.2	5	9 700	5 000 (52)
**16.2 Violence against children**			
16.2.1	1	3 880	3 793 (98)

SDG: sustainable development goal.

^a^ The text is an author summary of the text presented in Table 1.

^b^ Most subindicators have disaggregrations; examples include sex, age, unit of measurement, location and quartile as shown in Table 1.

^c^ This table presents results from the descriptive cross-sectional analysis to assess the percentage of missing data across the indicators. The analysis involved a Pearson correlation test between the number of disaggregations per indicator and the percentage of missing data, as explained in the methods section.

### Missing data by level of disaggregations

Table 2 shows that 72% (31/43) of indicators are disaggregated by at least one subindicator and in some cases include a combination of age, sex, unit of analysis (for example, percentage, absolute number or per population head) and/or other factors, as described in Table 1.

Indicators with multiple disaggregations showed similar missingness to those without. The correlation between disaggregation count and missing data is negligible (*r*: 0.08; 95% confidence interval: −0.23 to 0.37). For example, indicator 3.d.1, with 15 disaggregations, has 29% (8430/29 100) missing data, while indicator 3.3.4 (no disaggregations) has 85% (1652/1940) missing data, suggesting disaggregation does not explain missingness.

### Missing data by WHO region

**Fig. 1:**
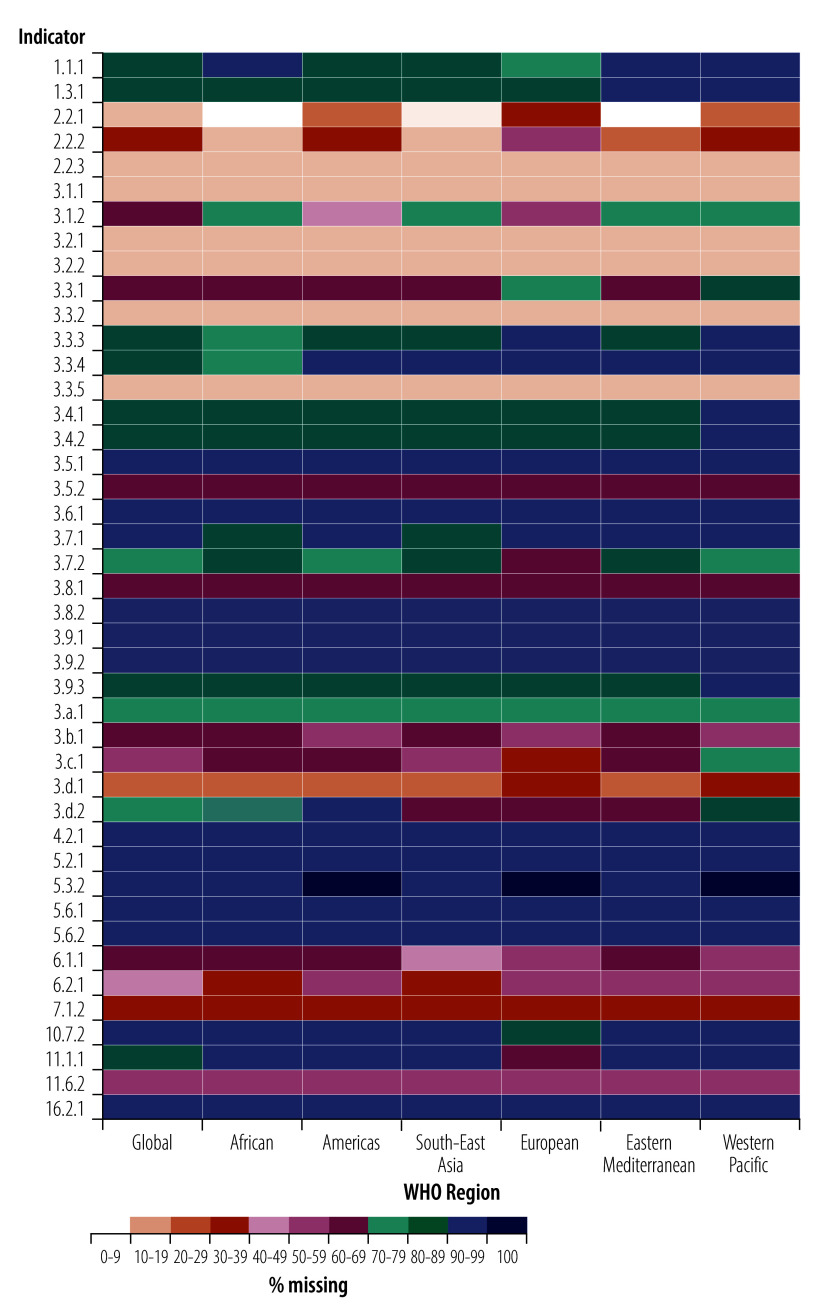
Distribution of missing health-related SDG data by WHO region

SDG: sustainable development goal; UN: United Nations; WHO: World Health Organization.

Notes: The data extracted for the analysis are the reported data of the 43 selected indicators in the UN SDG indicators database for all 194 WHO Member States, grouped by WHO region, 2015–2024. Examples of indicators and subindicators are: SDG 3.3 Communicable diseases, subindicator 3.3.1: Number of new HIV infections per 1000 uninfected population, by age and sex.

Source: United Nations Department of Economic and Social Affairs.^18^

Globally, a substantial portion of health-related SDG indicators exhibit high levels of missing data, with only a few indicators having relatively complete data points (Fig. 1). The Western Pacific Region has the highest percentage of missing data points, with 16 indicators missing more than 90% of data points, followed by the Eastern Mediterranean Region and the Region of the Americas in which 15 indicators are missing over 90% of data points. These regions are followed by the European Region, where 13 indicators are missing over 90% of data points. The African Region and South-East Asia Region have the least missing data, with 12 indicators missing more than 90% of data points. 

### Missing data over years

**Fig. 2:**
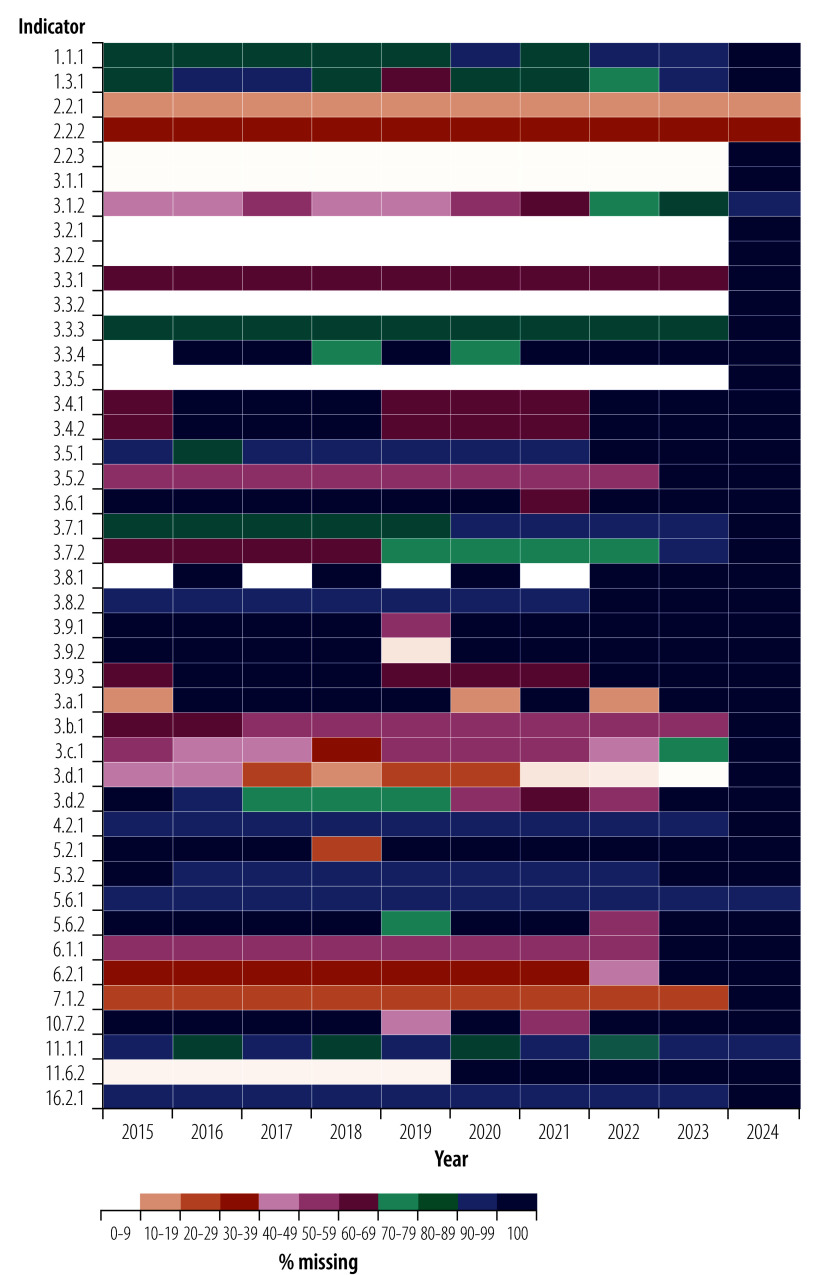
Distribution of missing health-related SDG data by year, 2015–2024

SDG: sustainable development goal; UN: United Nations; WHO: World Health Organization.

Notes: The data extracted for the analysis are the reported data of the 43 selected indicators in the UN SDG indicators database for all 194 WHO Member States for each year from 2015–2024.

Source: United Nations Department of Economic and Social Affairs.^18^

The patterns of missing data across years reveal significant inconsistencies in reporting for several indicators (Fig. 2). The year with the most indicators (41 out of 43) missing over 90% of data points was in 2024, while the year with the fewest (11 out of 43) was in 2019. Furthermore, indicator 11.6.2 maintained relatively low levels of missing data (10% or below) until 2020. Since 2020, no data have been available for this indicator, making recent trends untraceable. Additionally, four indicators (3.6.1, 3.9.1, 3.9.2 and 5.2.1) have data available for only a single year, providing a limited snapshot that prevents longitudinal analysis. 

### Missing data by method 

**Fig. 3:**
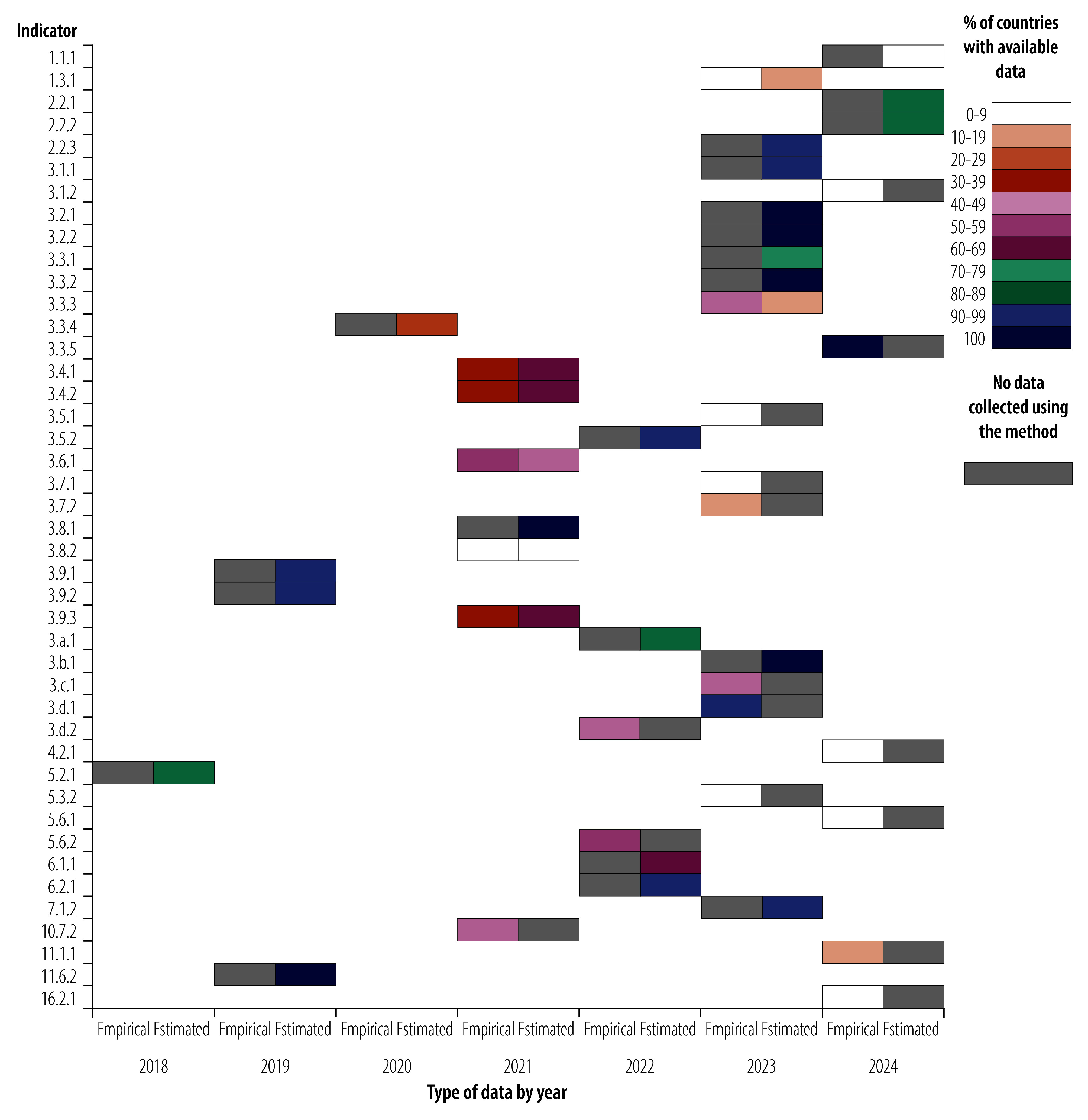
Proportion of countries with health-related SDG data by method of data collection for the most recent year

SDG: sustainable development goal; UN: United Nations; WHO: World Health Organization.

Notes: The data extracted for the analysis are the reported data of the 43 selected indicators from the UN SDG indicators database for all 194 WHO Member States for each year from 2015–2024 and according to the method of data collection. The plot on the left shows the most recent year with data for each indicator in any Member State. The figure presents data from 2019. For indicator 5.2.1, no shading is shown because the most recent available data year was 2018, before the cut-off year for inclusion in the figure. The plot on the right shows the share of Member States with data for the latest available year, disaggregated by empirical and estimated methods. The grey sections in the right-hand plot show indicators where no data were collected using the method.

Source: United Nations Department of Economic and Social Affairs.^18^

Among the 43 health-related indicators, the distribution between estimated and empirical data, as well as dual approach indicators (i.e. using both empirical and estimation methods simultaneously), reveals notable differences in the proportion of Member States with data for the most recent year for any single indicator. A total of 21 indicators (49%) rely solely on estimates, while 15 indicators (35%) are based solely on empirical data. The remaining 7 indicators (16%) use both empirical and estimated methods. Estimated indicators generally have a higher percentage of Member States with data for the most recent year than indicators relying on empirical data (Fig. 3).

## Discussion

Analysing the distribution of missing data for the health-related SDG indicators included in WHO’s GPW 14 across all 194 WHO Member States from 2015 to 2024 uncovered substantial gaps and inconsistencies. Although about three quarters of indicators included some form of disaggregation, as previously shown,^6^ substantial inconsistencies existed in the proportion of missing data in indicators disaggregated by age, sex and socioeconomic status, and no clear link between the level of disaggregation and the volume of missing data. Without adequate disaggregation, targeted interventions for vulnerable populations are harder to design and evaluate.^23^ Strengthening the collection, reporting and use of disaggregated data therefore requires greater investment, as articulated by WHO and other UN organizations, alongside efforts to understand factors affecting data availability, such as political will and infrastructure challenges.^9^^,^^11^

Variations in the proportion of missing data across countries and regions may be explained by several reasons. First, some indicators may address socially and politically sensitive issues, such as physical, sexual or psychological violence (indicator 5.2.1) and child physical and emotional abuse (indicator 16.2.1).^24^^,^^25^ Governments may also sometimes limit the reporting and public dissemination of such data.^6^ Additionally, some of these indicators are conceptually difficult to measure and are only available through large-scale surveys such as the Demographic and Health Surveys (DHS) and Multiple Indicator Cluster Surveys, which may be conducted infrequently, limiting their ability to capture short-term changes.^26^ Shifts in foreign policy, including potential reductions in funding for DHS or even their termination, highlight the reliance of many Member States on these surveys for tracking key indicators and the need to explore alternative data sources.^27^

The lack of data at different time points may be attributed to several factors. The high percentage of missing data in 2024 is expected, as the standard processing and reporting timeline of many indicators can take up to two years. Unlike certain international governing bodies to which countries have legal obligations to report data, WHO does not hold governing authority, making data submission a voluntary action. Formalized reporting agreements could significantly improve data availability, as evidenced by indicator 3.d.1 on IHR (2005) capacity and health emergency preparedness, which benefits from such an arrangement. Specifically, in 2008, States Parties agreed to report their implementation of IHR (2005) annually to the World Health Assembly using a globally standardized reporting form, resulting in the fewest data gaps for this indicator.^28^

Additionally, some data collection activities occur in rounds or multi-year cycles, such as the Childhood Obesity Surveillance Initiative or DHS. These data are collected at specified intervals and may be further complicated by unforeseen global events, such as pandemics, armed conflicts or extreme weather events. 

Estimated SDG data are more readily available than empirical data. To address outdated data, statistical modelling is used to produce estimates that complement empirical data, underscoring the need for standardized data-sharing practices.^6^^,^^15^ Such modelled estimates, which use information from time trends, neighbouring countries, socioeconomic covariates and past data to fill gaps, are especially useful when national data are unavailable.^29^

While the *Guidelines for accurate and transparent health estimates reporting* (GATHER) promote technical documentation for using diverse data sources and models, many global health estimation efforts are not yet fully compliant, and transparency remains constrained by limitations in data sharing and access.^6^ Strengthening GATHER to ensure transparency in data inputs, processing and analysis, including all health-related SDG-related data, as well as creating user-friendly outputs and maintaining trust in health institutions,^30^^–^^32^ is critical to enhance replicability and usability.^6^ Notably, using global estimates instead of national data can weaken country ownership and inadvertently create the impression that sufficient data already exists, discouraging investments in empirical data collection.^29^ Ultimately, data harmonization efforts remain vital to improve methods and enhance confidence in estimates, especially where discrepancies are created for indicators with limited original data.^15^^,^^33^^,^^34^

This study has several limitations. First, the focus on global and regional data from the UN SDGs indicators database may not fully represent data collection efforts within individual Member States. Second, the analysis primarily considers WHO Member States, and while territories or areas are not included, their data may offer additional insights. Third, we did not assess data quality aspects such as accuracy and reliability, particularly the challenges of ensuring a correct denominator and a high-quality numerator for the population under study, which may have influenced the results. Fourth, not all indicators are reported every year for all countries. Some indicators are only being reported biannually, while others have a more regional focus. Fifth, there is limited information about the type of calculation or the aim used to derive estimated data. For example, estimated data may be imputed or extrapolated from existing data, with limited details about the methods used. Sixth, the study’s reliance on descriptive analysis limits its ability to establish causal relationships or delve into the complex factors driving disparities in data availability. Seventh, the study assumed the number and definitions of disaggregations for each indicator remained the same over the study period. Effectively, new disaggregations added to each indicator would be counted as missing in the years before they were introduced.

Effective governance in global health requires accountability, which must be built on a foundation of timely, accurate and comprehensive data.^35^ This study highlights significant weaknesses in current data collection and reporting practices for health-related SDGs, leading to the following considerations: first, data coordination capacities at the multilateral and national levels, especially for health ministries and national statistical offices, must be enhanced through clear legal frameworks, data-sharing infrastructure and capacity-building. Stronger mechanisms for sharing technical expertise among WHO, other UN agencies, national statistical offices and ministries of health are also needed. Second, data timeliness and the absence of disaggregated data must be addressed as critical priorities by supporting standardized and regular data reporting, incentives and processes for country-level reporting, as well as accountability and contextual strategies for the collection and reporting of data on inequalities. Third, regular health information systems assessments must be implemented to identify political, technical and infrastructural constraints that lead to data gaps. Such constraints may then be reported and discussed transparently to co-create solutions and establish accountability for good data stewardship.

In the absence of reliable data, progress towards achieving the health-related SDGs is significantly undermined, thereby weakening accountability mechanisms and limiting the effectiveness of interventions. Prioritizing investment in robust data collection, comprehensive disaggregation and systematic reporting is not solely a technical consideration but a foundational element for promoting equity, transparency and meaningful progress in global health governance.
